# Design and Experimental Research of a Novel Stick-Slip Type Piezoelectric Actuator

**DOI:** 10.3390/mi8050150

**Published:** 2017-05-08

**Authors:** Mingxing Zhou, Zunqiang Fan, Zhichao Ma, Hongwei Zhao, Yue Guo, Kun Hong, Yuanshang Li, Hang Liu, Di Wu

**Affiliations:** School of Mechanical Science and Engineering, Jilin University, Changchun 130025, China; zhoumx15@mails.jlu.edu.cn (M.Z.); zcma@jlu.edu.cn (Z.M.); hwzhao@jlu.edu.cn (H.Z.); yueguo15@mails.jlu.edu.cn (Y.G.); hongkun15@mails.jlu.edu.cn (K.H.); ysli15@mails.jlu.edu.cn (Y.L.); liuhang15@mails.jlu.edu.cn (H.L.); wud15@mails.jlu.edu.cn (D.W.)

**Keywords:** piezoelectric, actuator, flexure hinge, stick-slip

## Abstract

A linear piezoelectric actuator based on the stick-slip principle is presented and tested in this paper. With the help of changeable vertical preload force flexure hinge, the designed linear actuator can achieve both large travel stick-slip motion and high-resolution stepping displacement. The developed actuator mainly consists of a bridge-type flexure hinge mechanism, a compound parallelogram flexure hinge mechanism, and two piezoelectric stacks. The mechanical structure and motion principle of the linear actuator were illustrated, and the finite element method (FEM) is adopted. An optimal parametric study of the flexure hinge is performed by a finite element analysis-based response surface methodology. In order to investigate the actuator’s working performance, a prototype was manufactured and a series of experiments were carried out. The results indicate that the maximum motion speed is about 3.27 mm/s and the minimum stepping displacement is 0.29 μm. Finally, a vibration test was carried out to obtain the first natural frequency of the actuator, and an in situ observation was conducted to investigate actuator’s stick-slip working condition. The experimental results confirm the feasibility of the proposed actuator, and the motion speed and displacement are both improved compared with the traditional stick-slip motion actuator.

## 1. Introduction

With the rapidly-growing demand for high accuracy in micro-nano fabrication and optical systems, designing high-accuracy nanopositioning systems has become more and more significant. Given the advantages of high-resolution, large output force, and rapid response, the piezoelectric nanopositioning stage has attracted widespread attention from researchers all over the world. In addition, many types of piezoelectric driven actuators have been successfully developed in the field of precision nanopositioning [1,2,3]. Regarding their working principles, piezoelectric actuators can be mainly divided into ultrasonic actuators, direct driving actuators, inchworm actuators, stick-slip actuators and so on. Nevertheless, all kinds of these piezoelectric-driven actuators have both advantages and disadvantages. Ultrasonic actuators have high-speed motion and rapid response, but the stability of the motion and heat generation are still problematic [4,5,6]. Direct-driving actuators have large output force and displacement and, furthermore, their structure can be compact and can achieve rapid responses with high resolutions; however, their operating stroke is limited [7,8,9]. Inchworm actuators have large motion ranges by stepping motions, and the output force is large, but the control system and mechanical structure are relatively complex [10,11,12]. A large amount of research still needs to be accomplished to overcome all of these actuators’ shortcomings and to develop new types of piezoelectric actuators. 

Nanopositioning actuators have been used in many applications. A number of applications need both nanoscale resolutions and large travel. Stick-slip actuators present the advantage to allow long displacements (several centimeters or even more) at a high speed (several mm/s) with an ultra-high resolution. Moreover, they are simple, compact, and offer a high stiffness. They are, therefore, perfectly well adapted to challenging applications, such as nanopositioning [13,14,15]. For example, Zhang et al. [16] presented a two-DOF (degree of freedom) rotary-linear actuator based on the stick-slip principle, which could achieve both linear displacement and angular displacement with the sequential coordination of three piezoelectric stacks. Nevertheless, when the coaxiality between actuator shaft and friction piece is not ideal, the vertically-distributed preload screw cannot provide a steady tightening force to balance the actuator shaft, implying that in order to achieve steady stick-slip motion, relatively high assembly accuracy is needed. Stieg et al. [17] proposed an inertial drive actuator for coarse and fine approaches in scanning probe microscopy, and it could perform clamping action by magnetic force without generating mechanical wear. However, the normal force between the slider element and drive body is constant, thus, the acceleration effects of gravity would lead to the slider element’s displacement loss in each working circle, so the stepping resolution and the stability of the stepping motion is limited. Li et al. [18] developed a stick-slip type linear actuator based on the lateral motion principle, it can realize fine linear motion by the flexure hinge’s parasitic movement, but there will be serious hysteresis and creep phenomena when the flexure hinge working under the high-frequency condition. So far, existing techniques in the design of piezo-driven stick slip actuators still cannot ensure high resolution, while meeting the requirements of achieving large stroke motion. Seldom have researchers paid attention to the unadjustable normal force between the mover and stator, which would restrict stepping resolution and motion speed of a stick-slip actuator to a great extent. 

Inspired by the above studies and the need for advanced stick-slip actuators, this paper proposes an actuator that can achieve both stable large-stroke and high-resolution linear motion, and the normal force between the stator and mover can be effectively adjusted with the help of adjusting an adjustable stage. The developed actuator mainly consists of a bridge-type flexure hinge mechanism, a compound parallelogram flexure hinge mechanism, and two piezoelectric stacks. Two kinds of working modes of this designed actuator are presented to realize different functions. When the piezo-stack that nested in the bridge-type flexure hinge mechanism works alone, the parasitic motion of the bridge-type flexure hinge mechanism enables the slider mover to achieve high-resolution fine motion, which can be applied in precise compensation for the position error. On the other hand, when two piezo-stacks that are nested in different flexure hinge mechanisms work together, the slider mover will achieve large stepping displacement motion due to the action of the compound parallelogram flexure hinge. This working mode can be brought into the adjustment of coarse positioning. This means the combination of two kinds of flexure hinge mechanisms enable the actuator to have a significant enhancement both in resolution of the stepping motion and the working stroke. 

Additionally, a series of experiments and finite element analysis methods were carried out to test the performance of the designed piezoelectric actuator. The proposed actuator may have some significance for designing stick-slip actuators and potential applications for a high-precision micro/nano-system. 

## 2. Design and Analysis

**Figure 1:**
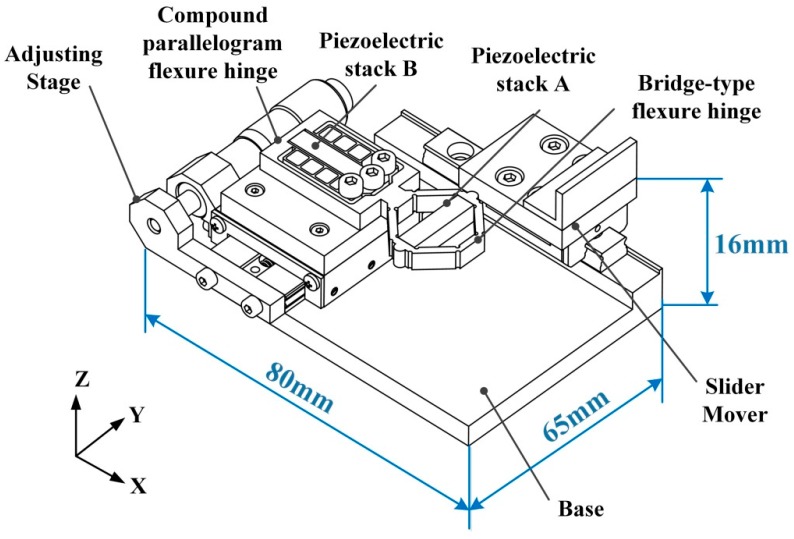
Mechanical structure of the designed actuator.

The structure of designed stick-slip piezoelectric actuator is illustrated in Figure 1. It can be clearly seen that the actuator mainly consists of a base, an adjusting stage, a slider mover, two piezoelectric stacks, a bridge-type flexure hinge, and a compound parallelogram flexure hinge. The adjusting stage (TSM13-2A from Zolix Company, Beijing, China) is made up of a linear spherical guide, an adjusting micrometer-knob and a moving platform. The flexure hinge mechanism is assembled on the top of the moving platform, and the moving platform is mounted on linear spherical guide. When turning the adjusting micrometer-knob, the fine motion of the moving platform is used to change preload normal force between slider mover and flexure hinge. Two piezoelectric stacks, nested in the bridge-type flexure hinge mechanism and a compound parallelogram, respectively, are exploited to deform the flexure hinge. A linear guide and a slider (SRS-9M from THK Company, Tokyo, Japan) are used as the mover for their high-carrying capacity and small-friction coefficient. All of the components are assembled on the base carefully. Since the assembling errors may influence the preload force, some adjustment work needs to be done to keep the working motion smooth after the assembly.

**Figure 2:**
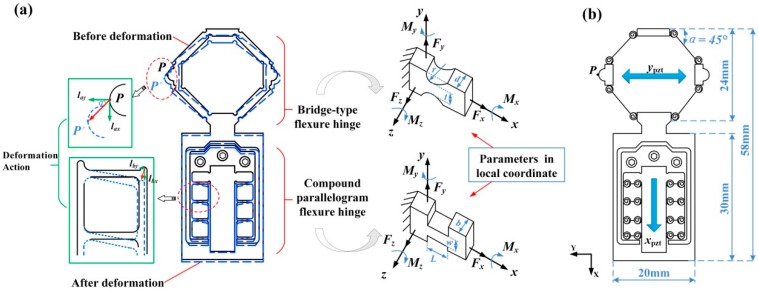
The designed flexure hinge: (**a**) structure; and (**b**) linkage model.

The geometric model of the bridge-type flexure hinge and compound parallelogram flexure hinge mechanism are shown in Figure 2. The key parameters of the designed flexure hinge mechanism are listed in Table 1. As the piezoelectric stack A receives power, it will deform the bridge-type flexure hinge both in *y* direction and *x* direction. The displacement *l_ay_* is the motion displacement generated along the elongation direction of piezoelectric stack A, The displacement *l_ax_* is lateral motion, and it is used to push the slider mover to move along the *x* direction. Additionally, when the piezoelectric stack B gets charged, it will extend along the *x* direction, then it pushes the compound parallelogram flexure hinge to deform both in the *x* and *y* directions, as well. However, due to the structural stiffness, the compound parallelogram flexure hinge will only deform a very small distance in the *y* direction, namely *l_by_*, and it can be ignored when compared with the large displacement in the *x* direction *l_ax_*. That is to say, the elongation of stack B can be entirely used to enlarge the stepping displacement of the slider mover in the *x* direction.

**Figure 3:**
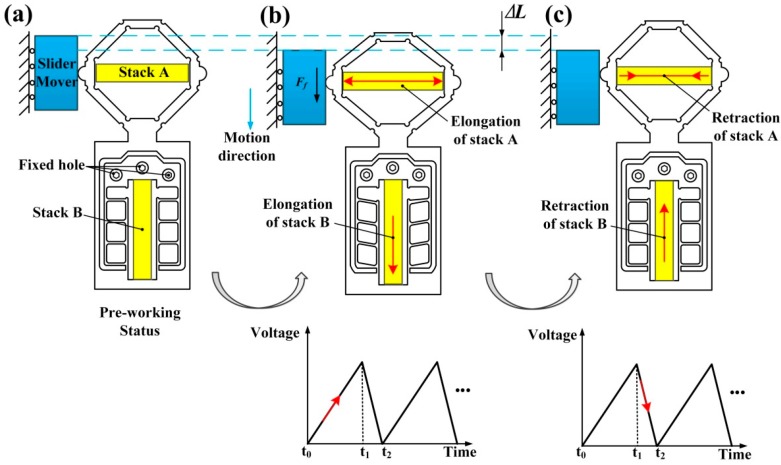
Working process of a circle: (**a**) the initial position; (**b**) the largest displacement position; and (**c**) the final position.

Based on the stick-slip principle, an asymmetrical sawtooth-wave voltage is applied to drive the piezoelectric stack, and the percentage of stacks’ charging time in a working circle is set to be 80%. The working process of a working circle about this designed actuator can be divided into two steps, as shown in Figure 3. From time *t*_0_ to *t*_1_, both piezoelectric stack A and B slowly obtain power, the piezoelectric stack A will push the bridge-type flexure hinge to move a certain distance both in the *x* and *y* directions, and the stack B extends slowly to push the compound parallelogram flexure hinge in the *x* direction as well. Since the bridge-type flexure hinge is holding the slider through the preload force, theoretically, the friction force between the slider mover and bridge-type flexure hinge is a static friction force, thus, under the deformation action of compound parallelogram flexure hinge, the piezoelectric stack B will push both the slider mover and piezoelectric stack A to move a small displacement ∆*L*, which is shown in Figure 3b; From time *t*_1_ to *t*_2_, piezoelectric stack A and B discharge quickly, resulting in the swift retraction of both the bridge-type flexure hinge and compound parallelogram flexure hinge to their initial position. The friction force between the flexure hinge and slider mover changes to be the sliding friction force. Nevertheless, due to the action of inertial force, the slider mover will stay nearly at the same position as in Figure 3b. Consequently, the slider moves forward one step displacement unit ∆*L* while the flexure hinge return to its initial position. As the sawtooth driving voltage increases and decreases cyclically, stick and slippage of the slider occur iteratively; hence, the slider is driven through an extended travel range, which is shown in Figure 3c.

Regarding the contact point P between the mover slider and bridge-type flexure hinge, the stepping displacement *x_p_* of point P can be considered as the sum of *l_ax_* and *l_bx_*:

(1) $$x_{p}=l_{ax}+l_{bx}$$

Here, *l_ax_* is the moving distance in *x* direction caused by piezoelectric stack A, and *l_bx_* is the moving distance in the *x* direction caused by the piezoelectric stack B. 

According to the previous works [19,20,21], the relationship between motion displacement of the bridge-type flexure hinge in the *x* and *y* directions can be obtained from the following equation:

(2) $$l_{ax}=\frac{y_{pzt}\cot\alpha }{2}$$

Here, *y_pzt_* is the elongation of piezoelectric stack A, and α denotes the inner angle of bridge-type flexure hinge, namely 45°.

Since the motion displacement in the *x* direction of piezoelectric stack B is fully used to push the slider mover, the moving distance caused by stack B can be written as follows, where *x_pzt_* represents the elongation of piezoelectric stack B:

(3) $$l_{bx}=x_{pzt}$$

As above mentioned, Equation (1) can be equivalent to the following:

(4) $$x_{p}=0.5y_{pzt}+x_{pzt}$$

The finite element analysis method (FEM) is adopted to investigate the working performance of the designed flexure hinge mechanism. The software ANSYS Workbench 15.0 is used to simulate in this study. The finest 3D tetrahedron element is used to mesh the model of the designed flexure hinge. Additionally, the piezoelectric stacks used are set as rigid bodies and the displacement controlled way is used for them. This is mainly due to the fact that the displacement of piezoelectric is approximately proportional to the driving voltage. When the driving voltage is 100 V, the elongation of piezoelectric stacks A and B from NEC is about 10 μm. Three screw holes are fully fixed during the simulation. The material used for the flexure hinge is Al 7075 and the main parameters are shown in Table 2.

**Figure 4:**
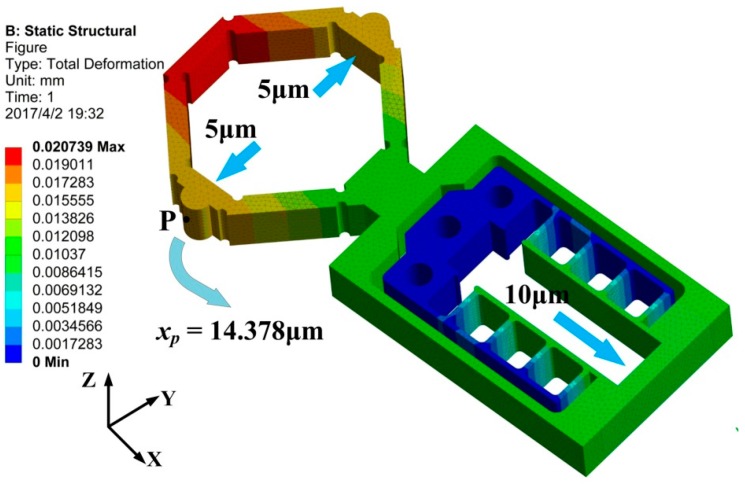
FEM result of the flexure hinge in the working direction.

The result of the FEM is illustrated in Figure 4, and it can be seen that the moving distance of point P in the *x* direction is 14.378 μm, which is nearly equal to the value obtained from Equation (4), namely 15 μm. Theoretically, the error may be due to the computational accuracy of the finite element method.

## 3. Optimization

In the past decades, structural optimization has been extensively explored and successfully applied to optimize structures and mechanisms in many practical engineering designs with the use of FEA [22]. The purpose of the optimization process for the flexure hinge mechanisms is to obtain optimal design parameters to achieve the maximum stepping displacement while taking into account of the stress concentration occurred in the flexural hinges. Generally, the radius *r*, thickness *w*, and length of rigid links *l* are the three important structural factors to the static performances of the flexure hinge, and these parameters are chosen as the design variables. Thus, the above multiple-objective optimization problem in this research is briefly described in the standard mathematical format as:

(5) $$Opt.\{\begin{array}{l} \mathrm{Find}:r,w,\;l\\ \mathrm{Maximize}\;\mathrm{displacement}\text{:}\;x_{p}\left(r,w,l\right)\\ \mathrm{Minimize}\;\mathrm{equivalent}\;\mathrm{stress}:S\left(r,w,l\right)\\ \mathrm{Subject}\;\mathrm{to}\;\mathrm{constrains}:\\ 0.54\leq r\leq \;0.66\\ 0.72\leq w\leq \;0.85\\ 3.6\leq l\leq \;4.2 \end{array}$$

**Figure 5:**
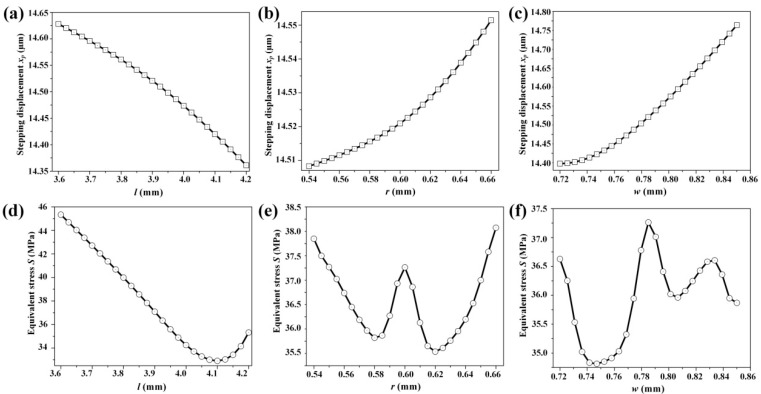
The dependence of the stepping displacement *x_p_* and maximum equivalent stress *S* on each of the design parameters: the change trend of stepping displacement with (**a**) *l*; (**b**) *r*; (**c**) *w*; and the change trend of maximum stress *S* with (**d**) *l*; (**e**) *r*; and (**f**) *w*.

**Figure 6:**
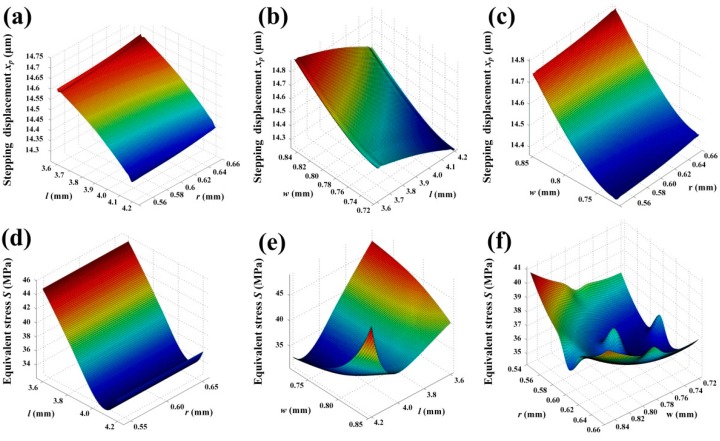
Stepping displacement *x_p_* plot in terms of (**a**) *l* and *r*, (**b**) *w* and *l*; (**c**) *r* and *w*; the maximum equivalent stress *S* plot in terms of (**d**) *l* and *r*; (**e**) *w* and *l*; and (**f**) *r* and *w*.

Since the response surface methodology (RSM) is a kind of mathematical and statistical technique that can be used to explore the relationships between several explanatory variables and one or more response variables [23], an optimization process is carried out in the commercial software ANSYS Workbench 15.0 to quickly reflect effects of different parameters on performance characteristics of the flexure hinge mechanism. Regarding the created response charts and surfaces, change trends of displacement and equivalent stress with designing parameters of flexure hinge mechanism are shown in Figure 5 and Figure 6. It can be noted that when *l* increases, the displacement and stress decrease slowly, as shown in Figure 5a,d. When *r* and *w* increase, their displacement quickly increase, as shown in Figure 5b,c. The stress will show slight increases when *r* increases, as shown in Figure 5e. Seen in Figure 6b,e, the sensitivities of both displacement and stress with *w* and *l* are considerately higher than with others. As illustrated in Figure 6a,c, *r* seems to have little influence on the displacement.

After computational analysis, the potential candidates are compared and estimated to find the best candidate, which can be seen in Table 3. According to Table 3, Candidate 1 was selected as the best optimal design because it completely satisfies the first design objectives: the maximum displacement *x_p_* was the highest among the candidates, and the maximum equivalent stress was still well below the yield strength of the material (505 MPa). Compared with the initial design, the static characteristics of the actuator was relatively improved, as seen in Table 3: (i) the stepping displacement was increased up to 2.99%; and (ii) the maximum stress was decreased by approximately 4.95%.

## 4. Experiment

### 4.1. Experimental Setup

**Figure 7:**
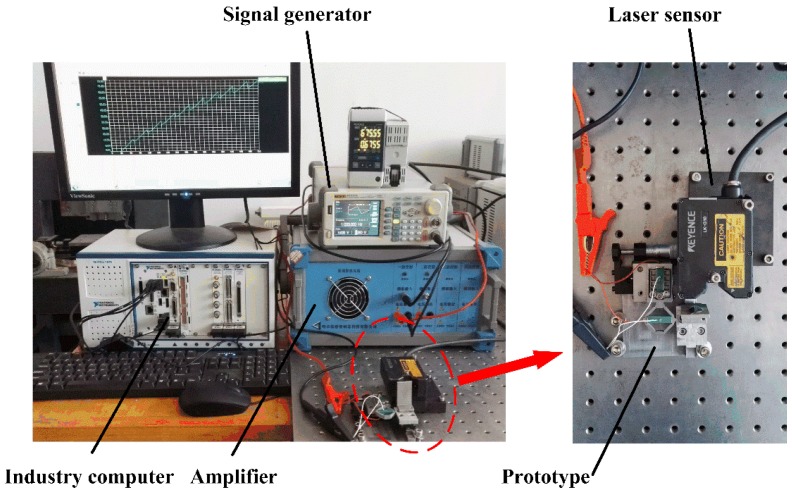
Experimental system and the prototype of the designed actuator.

A prototype of this stator has been constructed for the purpose of studying its performance, and the actuator possesses a compact structure with a whole size of 80 mm × 65 mm × 16 mm. Both the flexure hinge mechanism and the slider mover are fabricated from Al 7075 material due to its high elastic modulus and relatively larger stiffness. The prototype was manufactured by low-speed wire-cut electric discharge machining (WEDM) technique with the wire diameter being 0.4 mm, and the machining tolerances were specified as ±5 μm throughout. The experiment system is established as shown in Figure 7, and it mainly consists of one personal computer, one signal controller, one signal amplifier, one laser sensor, and the prototype. When the experimental system works, the signal generator DG3121A (from RIGOL Technologies, Beijing, China) will provide the needed sawtooth wave voltage signal, then the voltage signal will be amplified by the signal amplifier RH1500D/1 (from SOLUBLE CORE Technology, Harbin, China), the enlarged voltage is then used to drive the piezoelectric stacks. The maximum power of the RH1500D/1 amplifier (three channels) is 300 W. A laser sensor LK-G10 (from Keyence Corporation, Osaka, Japan) with a resolution of 10 nm is used to measure the moving displacement of the slider. The piezoelectric stack (AE0505D16) is from NEC-Tokin and the size is 5 mm × 5 mm × 20 mm. 

### 4.2. Motion Performance Tests

**Figure 8:**
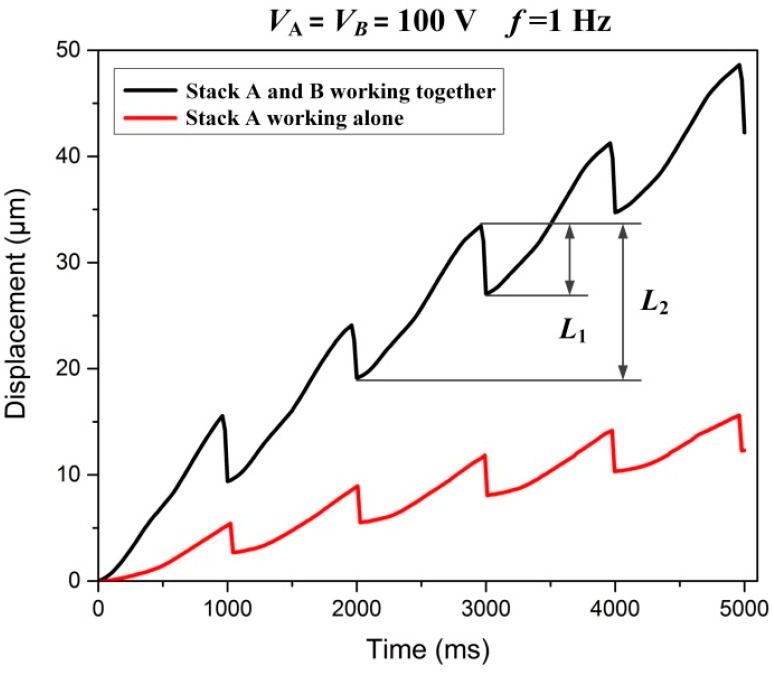
Motion displacement of two kinds working modes: stack A working alone, and stack A and B working together.

Figure 8 illustrates the comparison of motion performance about two kinds of working modes, namely stack A and B working together and stack A working alone. The given input voltages and driving frequency of two working modes are 100 V and 1 Hz, respectively. It can be seen that when the sawtooth wave voltage is applied to the piezoelectric stacks, the slider mover will move stepwise in a working circle, that is, even if the slider reaches the maximum displacement *L*_2_ at time *t*_1_, it will retract a slight distance *L*_1_ during time of *t*_2_. This may be caused by the condition that when flexure hinges reverting back to their undeformed shape, the slider is still in contact with the flexure hinge during time *t*_1_ to *t*_2_. Then the slider will move back a small distance *L*_1_ under the action of sliding friction force. Therefore, the real stepping displacement of the slider in a working circle can be calculated from the following equation:

(6) $$\Delta L=L_{2}-L_{1}$$

Here, ∆*L* is the stepping displacement, *L*_2_ is the maximum displacement of forward motion and *L*_1_ is the displacement of backward motion in a working circle. 

Additionally, it can be noted that the when stack A and stack B work together, the stepping displacement is obviously larger than that when stack A works alone. Seen from the Figure 8, when piezo stacks A and B work together at 100 V input voltage and 1 Hz driving frequency, the stepping displacement ∆*L* of the slider can reach 9.39 μm. Similarly, the stepping displacement is 2.68 μm when piezo stack A works alone. These results confirm that the combination of two kinds of flexure hinge mechanism enables the actuator to have a great enhance both in resolution of stepping motion and working stroke, and so does the motion speed of it, the motion speed can be written as follows:

(7) V=f×ΔL

where *V* is the motion speed of slider mover, *f* is the driving frequency, and ∆*L* is the stepping displacement in a working circle.

**Figure 9:**
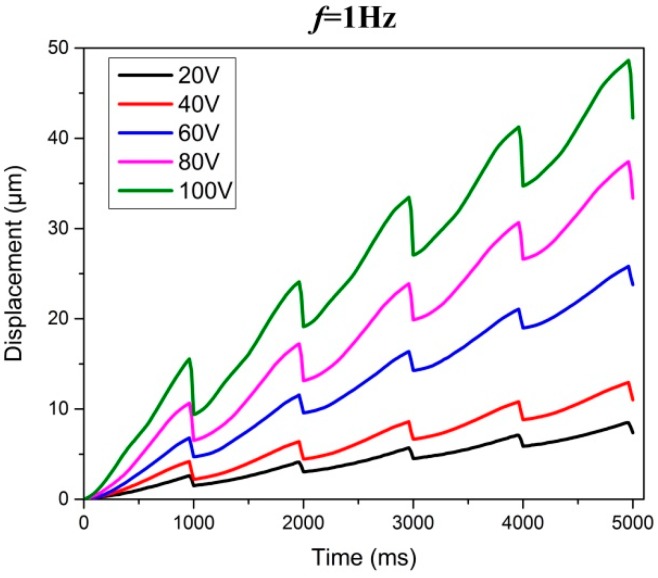
Motion displacement under different input voltages.

**Figure 10:**
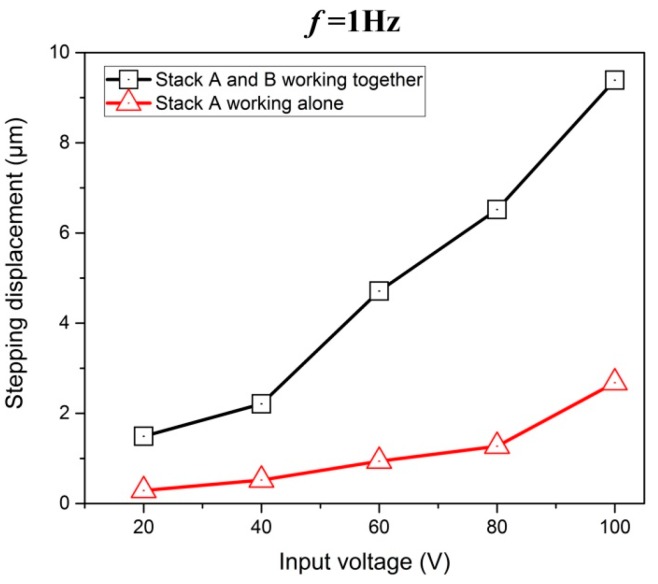
Stepping displacement under two working modes at different input voltages.

Figure 9 illustrates the motion displacement of the slider under different input voltages when stack A and stack B work together. Obviously, the motion displacement of the slider mover in one working circle varies with the input voltage. When the input frequency is relatively low, such as 1 Hz, the motion displacement will increase along with the input voltage stably. Figure 10 presents the relationship between stepping displacement and input voltage about two working modes. It can be clearly seen that the stepping displacement increases along with the enlargement of input voltage as well, and it agrees with the fact that the extension of piezoelectric stack will becomes greater when its driving voltage rises. The maximum stepping displacement is 9.37 μm when the stack A and stack B work together at 100 V input voltage. Considering the fact that when the input voltage is less than 20 V, the actuator cannot move forward smoothly, so the effective minimum stepping displacement of the actuator is 0.29 μm. That is, the motion resolution of this actuator can be equivalent to the effective minimum stepping displacement, namely 0.29 μm.

**Figure 11:**
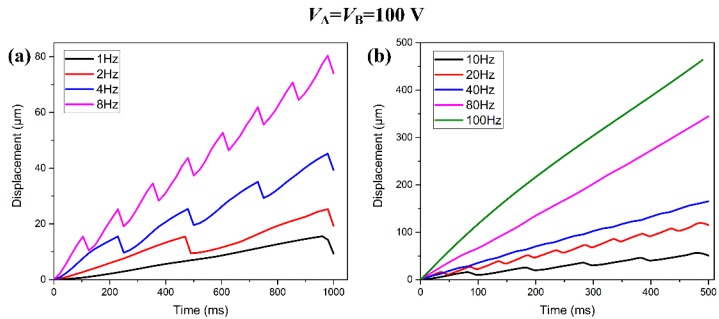
Motion displacement under different driving frequencies: (**a**) 1–8 Hz; and (**b**) 10–100 Hz.

As shown in Figure 11, when the input voltages of piezo stacks A and B are both 100 V, the motion displacement varies greatly with different driving frequency. Figure 11a illustrates the motion displacement of the slider when the piezo stacks are working under low frequency: 1, 2, 4, and 8 Hz. Figure 11b shows the motion displacement at the high frequency: 10, 20, 40, 80, and 100 Hz. These results indicate that, with the increases of driving frequency, the backward motion of the slider is more and more difficult to observe in a working circle. This is caused by the fact that the flexible part of the flexure hinge cannot recover to its undeformed shape in a limited response interval time before undergoing the next working circle. 

**Figure 12:**
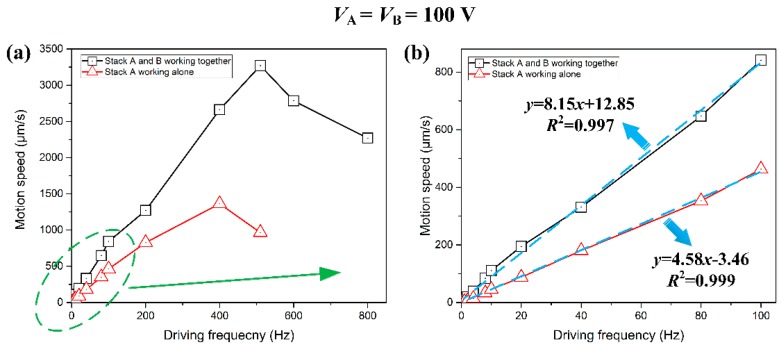
(**a**) Illustration of the motion speed under various driving frequencies; (**b**) illustration of the partial enlarged drawing of motion speed under low driving frequency: 1–100 Hz.

Accordingly, the motion speed is also considered to be another performance indicator of the designed actuator. Figure 12 presents the relationship between motion speed and driving frequency when the input voltages of stack A and stack B are both 100 V. It can be found in Figure 12a that the piezoelectric actuator would work stably when the driving frequency is below 510 Hz. However, the stepping displacement will sharply decrease when the driving frequency is higher than 510 Hz, and this may be caused by the fact that due to the flexure hinges’ mechanical inertia, the elastic deformation of flexure hinges cannot be synchronized with the elongation of stacks when the driving frequency is quite high. In other words, the piezoelectric stack is unable to accomplish a stable performance at fairly high driving frequency. According to the Equation (7), when the input voltage for both stack A and stack B is 100 V, along with the driving frequency being 510 Hz, the maximum motion speed of the slider is 3.27 mm/s. 

In order to quantitatively explain the correlation between motion speed and driving frequency, two linear equations of speed and driving frequency are given in Figure 12b by applying the least square method. Obviously, when the driving frequency is less than 100 Hz, the linear relationship can be approximately written as follows with the piezoelectric stacks A and B working together:

(8) y=8.15x+12.85

On the other hand, when piezoelectric stack A is working alone, the relationship can be approximately written as follows:

(9) y=4.58x−3.46

where *y* is the motion speed of the slider mover (μm/s), and *x* denotes the driving frequency (Hz).

In summary, the input voltage and driving frequency both can considerably influence the motion performance of the designed piezoelectric actuator. 

### 4.3. First Resonance Frequency Tests

**Figure 13:**
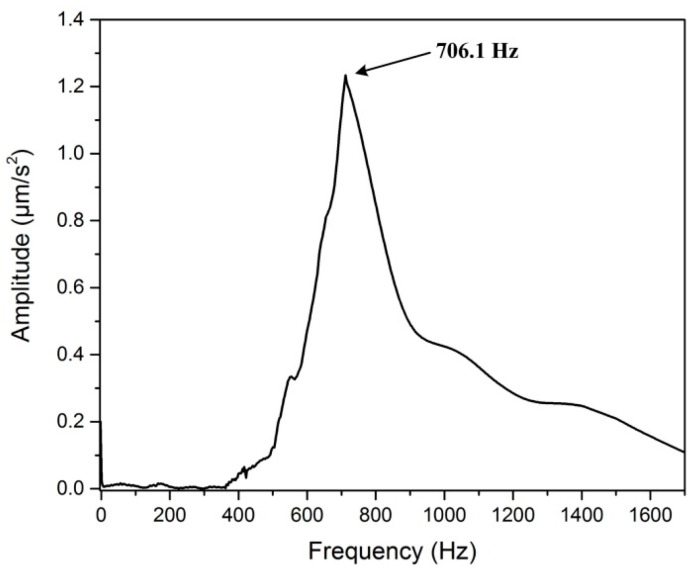
Experimental results of the first resonance frequency tests.

In order to verify that the stator will not show obvious vibration when it working under the low driving frequency, the first resonance frequency of the stator were measured by a vibration analyzer (INV3018C from China Orient Institute of Noise and Vibration). When conducting the test, a model hammer (Coinv MSC-3) is utilized to provide impulse force excitation, then the test data was collected and analyzed in a modal analyzer softer (Coinv DASP v10). Figure 13 illustrates the experimental result when the flexure hinge mechanism is actuated by the model hammer in the *x* direction. Similar results in the *y* direction can be obtained via the same method, but not illustrated herein. It can be found that the first resonance frequency of the designed actuator is 706.1 Hz, and there will be noticeable vibration only when the stator is operating at the relatively high driving frequency. That is, the stator can achieve stable linear motion when the piezo stacks are driven at the conventional testing frequencies.

### 4.4. Electrical Loading Rates Tests 

Electrical loading rates can considerably influence the responses of the structure with PZT actuators [24,25]. When the driving frequency and amplitude of the input voltage are unchangeable, the electrical loading rate is affected by the symmetry of sawtooth input voltage. Thus, five kinds of typical symmetry of this sawtooth wave voltage are applied to piezo stacks to compare the performance of the actuator.

**Figure 14:**
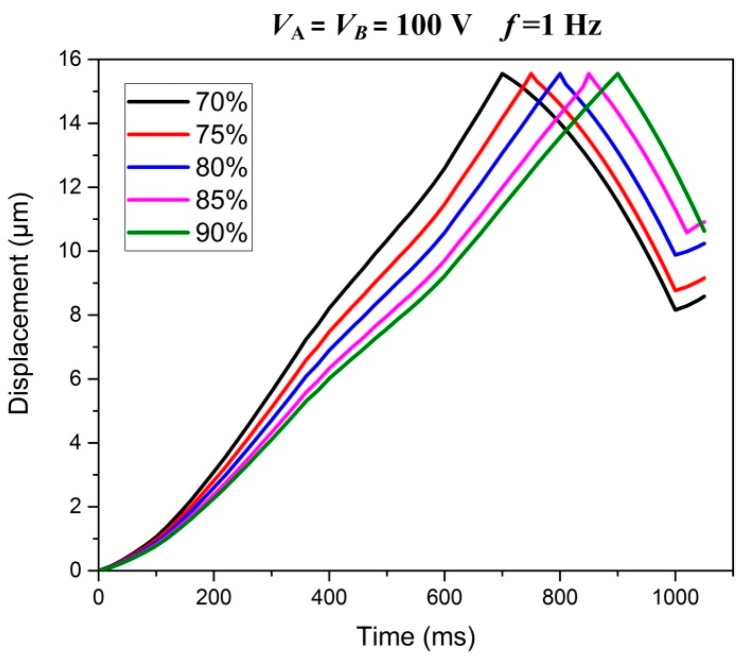
Displacement of the slider in a working circle with different percentages of stacks’ charging time.

Figure 14 illustrates the motion displacement of the slider in a working circle when piezo stacks are subjected to *V*_max_ = 100 (V) with different loading/unloading rates, and the driving frequency of the sawtooth wave voltage is 1 Hz. It can be clearly seen that when the percentage of the stacks’ charging time in a working circle is less than 80%, the retraction displacement of slider are significant, and it will seriously reduce the effective stepping displacement of the actuator. However, when the percentage of stacks’ charging time in a working circle is too high, for instance 85% or higher, the hysteresis of the displacement occurs more easily. That is to say, when the input voltage is abruptly unloading (*V*_max_→0), piezo stacks cannot be fully discharged due to their capacitance characteristics. 

### 4.5. In Situ Observation of the Stick-Slip Process

**Figure 15:**
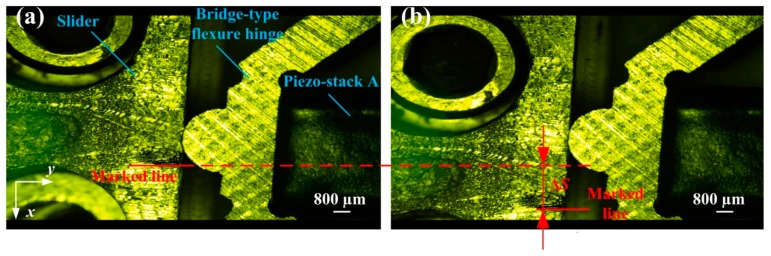
In situ observation of the stick-slip process: (**a**) initial position; and (**b**) ending position.

A charge-coupled device (CCD) camera (Nikon SMZ25, Tokyo, Japan) is used to record the stick-slip process in real-time. As shown in Figure 15a, a dark line is marked nearly to the contact region in the slider, and it is used as a reference to measure the motion displacement. Since the bridge-type flexure hinge is holding the slider through the preload force, it can push the slider to move along the *x* direction. By applying a continued 20 s driving voltage of 100 V and the driving frequency of 10 Hz sawtooth signal to the piezo-stacks, the piezoelectric stack B will push both the slider and piezo-stack A to move a large-stroke displacement ∆*S* of 2.04 mm, as shown in Figure 15b. It is verified that the combination of two kinds of flexure hinges and an adjustable-normal-force mechanism gains the advantage in enhancing working stroke for the nanopositioning systems. Moreover, for a video of the in situ observation of the stick-slip please see the Supplementary Information.

## 5. Conclusions

A novel piezo-driven linear actuator based on the stick-slip principle is proposed in this paper. It can achieve both large-stroke and high-resolution linear motion by using only two piezo stacks. The actuator mainly consists of a bridge-type flexure hinge, a compound parallelogram flexure hinge, and a linear motion module. The working principle and optimization of geometry are discussed. To verify the working performance of the actuator, a prototype is fabricated and a series of experiments are conducted. Furthermore, the variations in step sizes and velocities are observed by changing the driving voltage and frequency, respectively. The minimum step size for the linear motion is 0.29 μm when the input voltage is 20 V. Moreover, the actuator has maximum linear velocity of 3.27 mm/s when all driving voltages and the frequency are 100 V and 510 Hz, respectively. The experiment results confirm that the combination of two kinds of flexure hinges and an adjustable-normal-force mechanism is valid in enhancing positioning accuracy and working stroke for nanopositioning systems. Further studies will be performed to investigate the relationship between stepping displacement and roughness of the contacting surface in the future.

## Figures and Tables

**Table 1:** Key parameters of bridge-type flexure hinge and the compound parallelogram flexure hinge.

Parameters	*α*	*r*	*b*	*w*	*t*	*d*	*l*
Value	45°	0.6 mm	6 mm	0.8 mm	0.8 mm	6 mm	4 mm

**Table 2:** Mechanical properties of the material used in the FEM.

Parameter	Mechanical Properities
Young’s modulus *E*	72,000 (MPa)
Poisson’s ratio *ν*	0.33
Yield stress *σ_s_*	505 (MPa)
Density *ρ*	2810 (kg/m^3^)

**Table 3:** Comparisons among candidates.

Candidate Points	*l* (mm)	*w* (mm)	*r* (mm)	Displacement *x_p_* (μm)	Stress *S* (MPa)	Improvement of *x_p_* and *S*
Candidate 1	3.6765	0.8491	0.5909	14.85	38.56	2.99%, 4.95%
Candidate 2	3.7341	0.8473	0.6107	14.63	37.82	1.75%, 6.78%
Candidate 3	3.7917	0.8482	0.6054	14.79	39.18	2.86%, 3.42%

## Supplementary Materials

The following are available online at www.mdpi.com/2072-666X/8/5/150/s1. Video S1: In situ observation of the stick-slip process in the CCD camera.

## References

[1] Yong Y.K., Moheimani S.O.R., Kenton B.J., Leang K.K. (2012). Invited Review Article: High-speed flexure-guided nanopositioning: Mechanical design and control issues. Rev. Sci. Instrum..

[2] Tian Y., Zhang D., Shirinzadeh B. (2011). Dynamic modelling of a flexure-based mechanism for ultra-precision grinding operation. Precis. Eng..

[3] Polit S., Dong J. (2011). Development of a High-Bandwidth XY Nanopositioning Stage for High-Rate Micro-/Nanomanufacturing. IEEE/ASME Trans. Mechatron..

[4] Hou X., Lee H.P., Ong C.J., Lim S.P. (2013). Development and numerical characterization of a new standing wave ultrasonic motor operating in the 30–40 kHz frequency range. Ultrasonics.

[5] Lim K.J., Lee J.S., Park S.H., Kang S.H., Kim H.H. (2007). Fabrication and characteristics of impact type ultrasonic motor. J. Eur. Ceram. Soc..

[6] Fernandez J.M., Perriard Y. Comparative analysis and modeling of both standing and travelling wave ultrasonic linear motor. Proceedings of the 2003 IEEE Symposium on Ultrasonics.

[7] Park K.H., Lee J.H., Kim S.H., Kwark Y.K. (1995). High speed micro positioning system based on coarse/fine pair control. Mechatronics.

[8] Wu Z., Li Y. (2014). Design, Modeling, and Analysis of a Novel Microgripper Based on Flexure Hinges. Adv. Mech. Eng..

[9] Li C., Gu G., Zhu L., Su C. (2016). Odd-harmonic repetitive control for high-speed raster scanning of piezo-actuated nanopositioning stages with hysteresis nonlinearity. Sens. Actuators A.

[10] Park J., Keller S., Carman G.P., Hahn H.T. (2001). Development of a compact displacement accumulation actuator device for both large force and large displacement. Sens. Actuators A.

[11] Peng Y., Peng Y., Gu X., Wang J., Yu J. (2015). A review of long range piezoelectric motors using frequency leveraged method. Sens. Actuators A.

[12] Den Heijer M., Fokkema V., Saedi A., Schakel P. (2014). Improving the accuracy of walking piezo motors. Rev. Sci. Instrum..

[13] Chen T., Wang Y., Yang Z., Liu H., Liu J., Sun L. (2017). A PZT Actuated Triple-Finger Gripper for Multi-Target Micromanipulation. Micromachines.

[14] Breguet J.-M., Clavel R. Stick and slip actuators: Design, control, performances and applications. Proceedings of the 1998 International Symposium on Micromechatronics and Human Science (1998 MHS’98).

[15] Pan P., Yang F., Wang Z., Zhong B., Sun L., Ru C. (2016). A Review of Stick—Slip Nanopositioning Actuators. Nanopositioning Technologies.

[16] Zhang Y., Zhang W., Hesselbach J., Kerle H. (2006). Development of a two-degree-of-freedom piezoelectric rotary-linear actuator with high driving force and unlimited linear movement. Rev. Sci. Instrum..

[17] Stieg A.Z., Wilkinson P., Gimzewski J.K. (2007). Vertical inertial sliding drive for coarse and fine approaches in scanning probe microscopy. Rev. Sci. Instrum..

[18] Li J., Zhou X., Zhao H., Shao M., Hou P., Xu X. (2015). Design and experimental performances of a piezoelectric linear actuator by means of lateral motion. Smart Mater. Struct..

[19] Hao G., Kong X. Conceptual Design and Modelling of a Self-Adaptive Compliant Parallel Gripper for High-Precision Manipulation. Proceedings of the ASME 2012 International Design Engineering Technical Conferences and Computers and Information in Engineering Conference.

[20] Hao G., Hand R.B. (2016). Design and static testing of a compact distributed-compliance gripper based on flexure motion. Arch. Civ. Mech. Eng..

[21] Hao G., Li H. (2015). Nonlinear Analytical Modeling and Characteristic Analysis of a Class of Compound Multi-beam Parallelogram Mechanisms. J. Mech. Robot..

[22] Hao G., Kong X., Reuben R.L. (2011). A nonlinear analysis of spatial compliant parallel modules: Multi-beam modules. Mech. Mach. Theory.

[23] Khuri A.I., Mukhopadhyay S. (2010). Response surface methodology. Wiley Interdiscip. Rev. Comput. Stat..

[24] Hunstig M. (2017). Piezoelectric Inertia Motors—A Critical Review of History, Concepts, Design, Applications, and Perspectives. Actuators.

[25] Luo Q., Tong L. (2015). Design and testing for shape control of piezoelectric structures using topology optimization. Eng. Struct..

